# General Anesthesia for Emergency Cesarean Section in a Parturient With Previously Undiagnosed Neurofibromatosis Type 1: A Case Report

**DOI:** 10.7759/cureus.110387

**Published:** 2026-06-07

**Authors:** Youssra Kramchi, Yasser Ouatab, Nabil Elachhab, Sophia Lahbabi, Rajae Tachinante

**Affiliations:** 1 Anesthesia and Intensive Care, Mohammed V Military Training Hospital, Rabat, MAR; 2 Maternal Critical Care and Anesthesiology, Centre Hospitalo-Universitaire (CHU) Ibn Sina, Rabat, MAR; 3 Anesthesia and Critical Care, Centre Hospitalo-Universitaire (CHU) Ibn Sina, Rabat, MAR

**Keywords:** cesarean section, difficult airway, emergency obstetrics, general anesthesia, neurofibromatosis type 1, rapid sequence induction, rocuronium, undiagnosed neurofibromatosis

## Abstract

Neurofibromatosis type 1 (NF1) is an autosomal dominant multisystem disorder that presents significant challenges to the anesthesiologist, particularly in the obstetric setting. The presence of neurofibromas along the airway, potential spinal and intracranial tumors, cardiovascular instability, and altered pharmacological responses collectively complicate anesthetic planning. We report the case of a 42-year-old primigravida at 40 weeks and two days of gestation with previously undiagnosed NF1, admitted for emergency cesarean section under the clinical suspicion of uterine rupture. General examination revealed extensive cutaneous lesions and bilateral breast masses consistent with cutaneous and plexiform neurofibromas. Airway assessment identified a Mallampati grade III classification. Given the strong clinical suspicion of NF1, the absence of prior neuroimaging, and the emergency nature of the procedure, general anesthesia was chosen over neuraxial anesthesia. Rapid sequence induction was performed with propofol and rocuronium, with full difficult airway equipment immediately available. Direct laryngoscopy yielded a Cormack-Lehane grade I view with no glottic lesions identified, and orotracheal intubation was accomplished without difficulty. Anesthesia was maintained with isoflurane in an oxygen/air mixture (fraction of inspired oxygen (FiO2) 0.4). The procedure lasted 45 minutes, hemodynamic stability was maintained throughout without vasopressor support, and a healthy female neonate was delivered with Apgar scores of 9, 10, and 10 at one, five, and 10 minutes. Emergence from anesthesia was smooth and uneventful. This case highlights the importance of systematic clinical recognition of NF1 at admission, thorough airway evaluation, appropriate anesthetic decision-making in the absence of neuroimaging, and the critical role of anticipatory preparation in emergency obstetric scenarios involving undiagnosed multisystem disease.

## Introduction

Neurofibromatosis type 1 (NF1), also known as von Recklinghausen's disease, is one of the most common autosomal dominant genetic disorders, affecting approximately one in 3500 individuals worldwide [1]. It is caused by a mutation in the NF1 gene located on chromosome 17q11.2, which encodes neurofibromin, a tumor suppressor protein involved in the regulation of Ras-mediated cellular proliferation [1,2]. The disorder is characterized by multisystem involvement, with hallmark cutaneous features including café-au-lait macules, cutaneous neurofibromas, and axillary or inguinal freckling, alongside the potential involvement of the nervous system, cardiovascular system, respiratory system, and musculoskeletal system [3].

The anesthetic management of patients with NF1 is inherently complex. Neurofibromas involving the tongue, pharynx, larynx, and supraglottic region may cause airway obstruction or render intubation difficult or impossible, with reported cases requiring emergency tracheostomy [4]. The potential presence of clinically silent spinal neurofibromas (detected on MRI in up to 40% of asymptomatic NF1 patients in a referral cohort [5]) poses a significant risk when neuraxial anesthesia is considered. Additional concerns include cardiovascular instability from occult pheochromocytoma or renovascular hypertension, potentially altered sensitivity to neuromuscular blocking agents, and respiratory compromise from kyphoscoliosis or pulmonary fibrosis [4].

These challenges are further compounded in the obstetric setting. Pregnancy is associated with an increase in the number and size of neurofibromas, a higher incidence of hypertension and HELLP (hemolysis, elevated liver enzymes, and low platelets) syndrome, elevated rates of spontaneous abortion and preterm labor, and the physiological airway changes that independently increase the risk of failed intubation [3,4]. Crucially, no standardized anesthetic guidelines exist for the management of NF1 in pregnancy, and neither neuraxial nor general anesthesia can be considered inherently safe in this population. The choice between them depends entirely on pre-procedural imaging and the systematic evaluation of lesion burden, airway anatomy, and cardiovascular status. When NF1 has not been previously diagnosed, as in our case, the anesthesiologist must make rapid decisions in the absence of neuroimaging or systemic workup, relying entirely on clinical evaluation performed under time pressure.

Despite a growing body of case reports addressing NF1 in the obstetric context, the literature remains largely descriptive, with no consensus on anesthetic conduct, and cases involving previously undiagnosed NF1 presenting as an obstetric emergency remain rare. We present the case of a 42-year-old primigravida with previously undiagnosed NF1 who underwent emergency cesarean section under general anesthesia, with detailed attention to our clinical reasoning, airway assessment, anesthetic conduct, and perioperative course.

## Case presentation

Patient and admission

A 42-year-old primigravida (weight 60 kg, height 156 cm, BMI 24.7 kg/m²) at 40 weeks and two days of gestation by ultrasound dating was admitted to our institution for emergency cesarean section. The surgical indication was clinical suspicion of uterine rupture, based on the presence of uterine hypertonicity and severe hyperalgesia on palpation in the context of a term primigravida. Cardiotocographic monitoring was reassuring, with no evidence of fetal compromise. While the clinical presentation was not fully typical of classical uterine rupture, the obstetrician deemed the findings sufficiently concerning to preclude expectant management, and the procedure was classified as an immediate obstetric emergency, precluding pre-anesthetic imaging or systematic workup.

Her documented medical history included a total thyroidectomy performed four years prior, with subsequent hypothyroidism managed with levothyroxine 100 micrograms daily. Recent thyroid function tests, brought by the patient at admission, confirmed euthyroid status on her current regimen. This was her first pregnancy. She had no known drug allergies and no personal or family history of neurofibromatosis or any neurocutaneous disorder. No prior medical records documented a diagnosis of NF1.

Recognition of NF1

**Figure 1:**
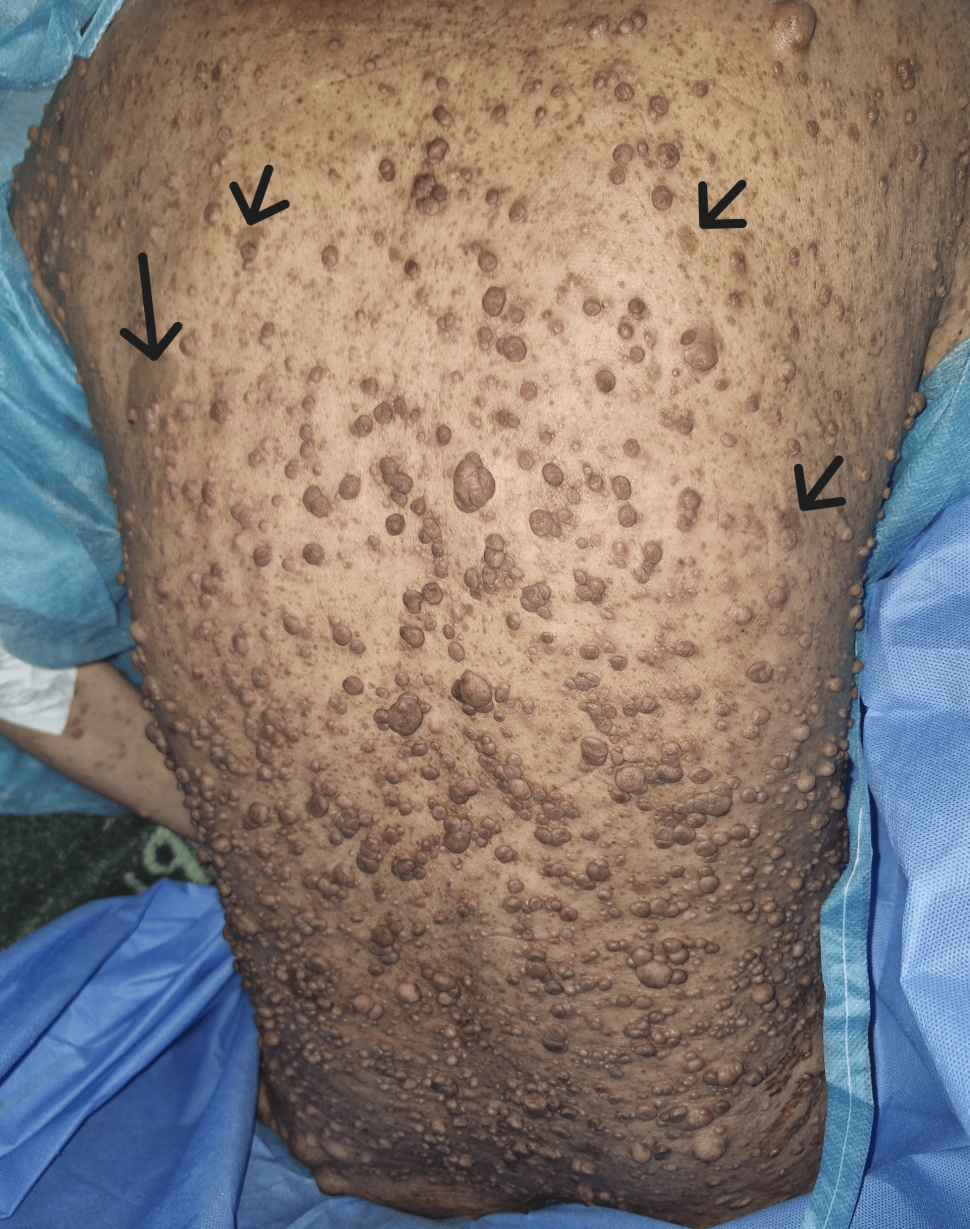
Extensive cutaneous neurofibromas and café-au-lait macules on the parturient's back. Posterior view of the parturient's trunk demonstrating the dermatological hallmarks of neurofibromatosis type 1. Numerous dome-shaped cutaneous neurofibromas of varying size are shown, and multiple flat, hyperpigmented areas consistent with café-au-lait macules (arrows) are visible as interspersed between the neurofibromas.

On physical examination performed at admission, the anesthesiology team identified extensive cutaneous manifestations highly consistent with NF1. The skin examination revealed diffuse café-au-lait macules distributed across the trunk and extremities, alongside hundreds of soft, dome-shaped cutaneous neurofibromas of varying sizes scattered across the entire body surface [6-8] (Figure 1).

**Figure 2:**
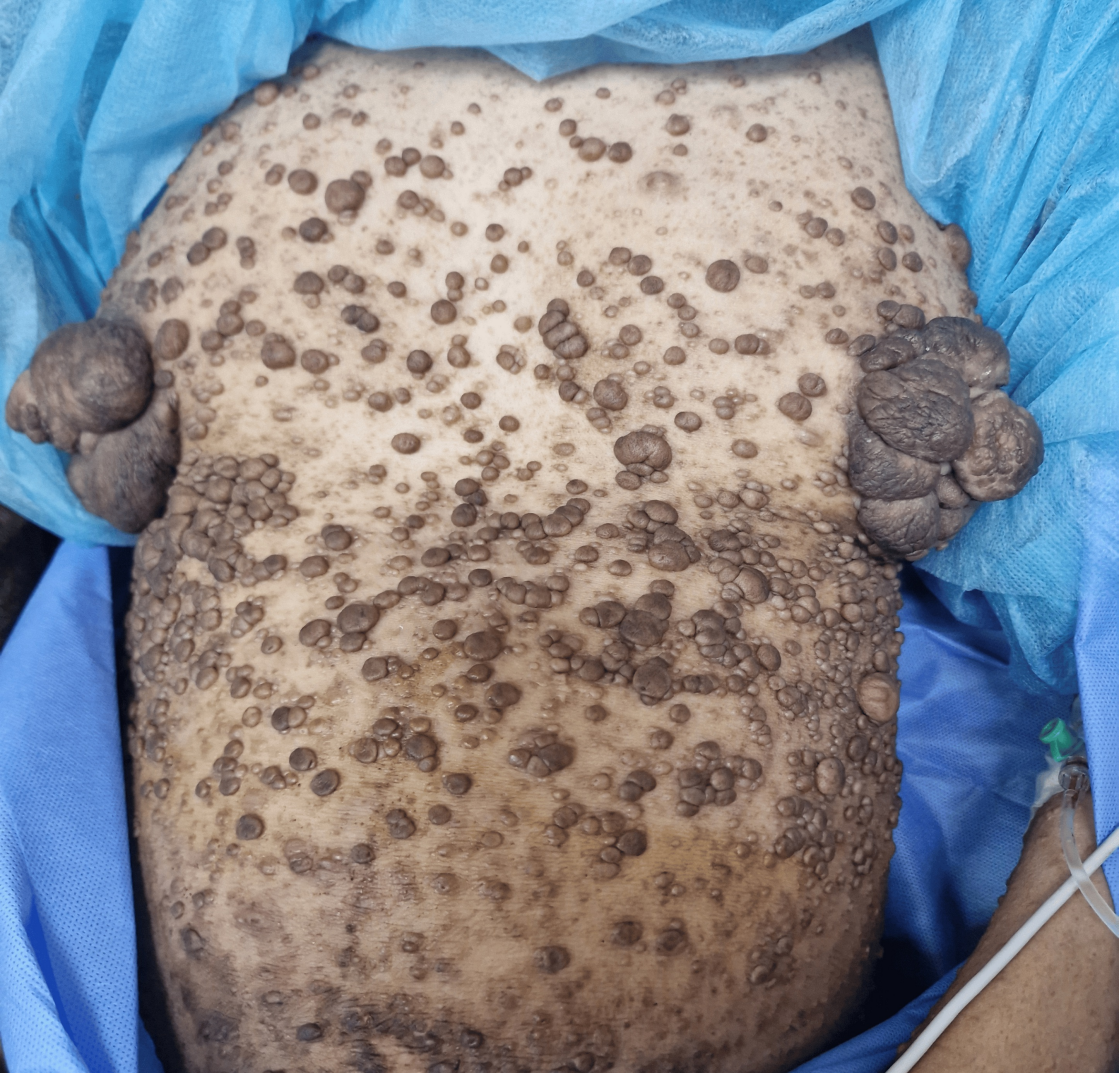
Diffuse cutaneous neurofibromas of varying sizes with bilateral large lobulated masses involving the nipple-areola region.

The overall tumor burden was considerable, with cutaneous lesions of varying sizes distributed extensively across the body surface. Of particular note was the bilateral involvement of the breast and nipple-areolar complexes by large lobulated masses, morphologically distinct from the surrounding cutaneous neurofibromas by virtue of their disproportionate size, irregular lobulated architecture, and anatomical distribution consistent with peripheral nerve trajectories. These features raised the possibility of plexiform involvement, which could not be histopathologically confirmed (Figure 2).

Plexiform neurofibromas are benign but infiltrating tumors that arise along the course of a nerve and its branches; they can involve deep anatomical structures and carry a lifetime risk of malignant transformation into malignant peripheral nerve sheath tumors (MPNST), estimated at 2-10% in NF1 patients [9]. As formal pathological or radiological confirmation was not possible in the emergency context, these lesions are described as consistent with, rather than confirmed, plexiform neurofibromas. Their presence raised concern for possible internal involvement along the airway, mediastinum, or spinal axis, with direct implications for anesthetic management.

**Figure 3:**
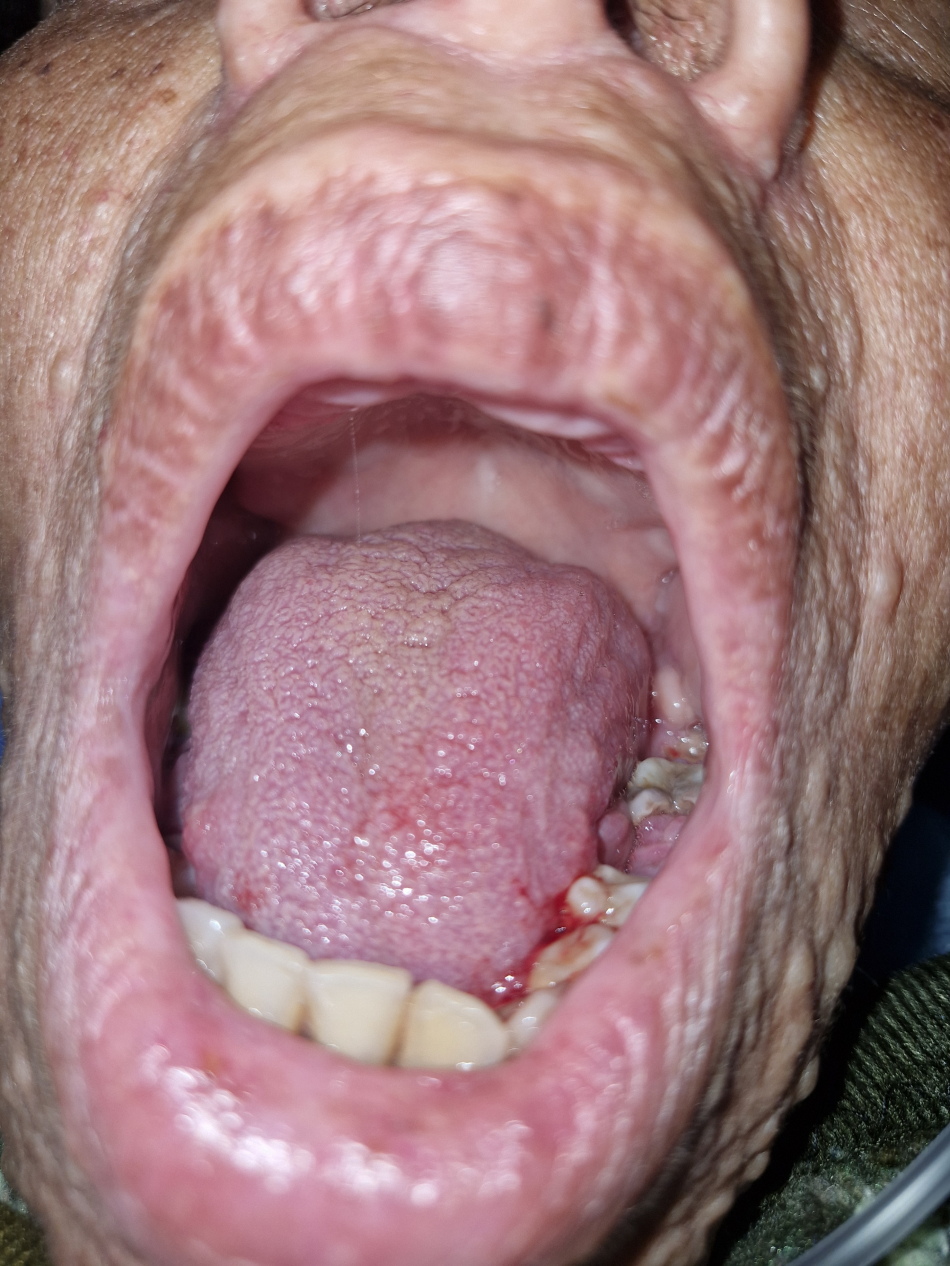
Oral cavity examination demonstrating perioral cutaneous neurofibromas.

Oral cavity examination was unremarkable, although extensive perioral cutaneous neurofibromas were noted (Figure 3). These lesions were carefully noted as relevant to airway planning, given the well-documented risk of supraglottic and laryngeal neurofibroma involvement in NF1 patients.

A dermatology consultation was promptly obtained at admission and concurred with the clinical diagnosis of NF1. The patient fulfilled at least two of the National Institutes of Health (NIH) diagnostic criteria [10]: multiple café-au-lait macules and numerous cutaneous neurofibromas. These criteria, established in 1988 [10], have since been revised to incorporate updated clinical features and genetic testing [11]; the patient also fulfilled the 2021 revised criteria based on cutaneous findings alone. Interestingly, the patient had no prior awareness of this diagnosis. No family history of neurofibromatosis was elicited, suggesting a de novo mutation, which accounts for approximately 50% of NF1 cases [1,2].

Anesthetic assessment and planning

Given the emergency nature of the procedure and the complete absence of prior neuroimaging, the decision was made to proceed with general anesthesia. This decision was based on the established principle that neuraxial anesthesia carries a significant risk in NF1 patients when spinal or intracranial neurofibromas cannot be excluded by preoperative imaging [4]. Performing spinal or epidural anesthesia in the presence of uncharacterized intracranial or spinal tumors risks direct mechanical injury to a spinal lesion, epidural hematoma, or, in the case of unrecognized raised intracranial pressure, cerebral herniation following dural puncture [4].

Airway assessment was performed systematically. The patient had a Mallampati grade III classification, a thyromental distance of 6 cm, normal mouth opening, and unrestricted neck mobility. The oral cavity itself appeared unremarkable; however, the possibility of supraglottic or glottic involvement could not be excluded without endoscopic evaluation, which was not feasible in this emergency context.

The full difficult airway cart was prepared and immediately available at the bedside, including a video laryngoscope, supraglottic airway devices, and equipment for surgical airway access. Succinylcholine was not available at our institution; rocuronium was selected as the neuromuscular blocking agent, with sugammadex immediately available for reversal if required. The NF1 literature documents potentially increased sensitivity to non-depolarizing neuromuscular blocking agents [4,7], and while neuromuscular monitoring was unavailable in our setting, this pharmacological consideration informed our vigilance during emergence.

Preoperative vital signs were as follows: arterial blood pressure 120/70 mmHg, heart rate 90 beats per minute, temperature 37°C, and peripheral oxygen saturation 98% on room air. A summary of patient characteristics and perioperative data is provided in Table 1.

**Table 1:** Patient characteristics and perioperative data. NF1: neurofibromatosis type 1; NIH: National Institutes of Health; SpO2: saturation of peripheral oxygen; PACU: post-anesthesia care unit; FiO2: fraction of inspired oxygen

Parameter	Detail
Patient demographics	
Age	42 years
Weight/height/BMI	60 kg/156 cm/24.7 kg/m²
Gestational age	40 weeks + 2 days
Parity	Primigravida (G1P0)
Medical history	
NF1 status at admission	Previously undiagnosed; established clinically at admission
Other conditions	Total thyroidectomy (4 years prior); hypothyroidism
Current medication	Levothyroxine 100 mcg/day
Thyroid function at admission	Normal (recent laboratory confirmed euthyroid)
Family history of NF1	None elicited
NF1 clinical findings	
Café-au-lait macules	Present
Cutaneous neurofibromas	Present, extensive, whole body surface
Plexiform neurofibromas	Suspected, bilateral breast/nipple-areolar complex (morphological assessment only; histopathological confirmation not obtained)
NIH diagnostic criteria fulfilled	At least 2 (café-au-lait macules + neurofibromas)
Preoperative vital signs	
Arterial blood pressure	120/70 mmHg
Heart rate	90 bpm
Temperature	37°C
SpO2 (room air)	98%
Airway assessment	
Mallampati grade	III
Mouth opening	Normal
Thyromental distance	6 cm
Neck mobility	Normal
Difficult airway cart	Prepared and immediately available
Surgical details	
Indication	Suspected uterine rupture (not confirmed intraoperatively)
Intraoperative finding	Uterine wall intact, no dehiscence
Surgery duration	45 minutes
Estimated blood loss	Minimal, less than 500 ml
Anesthetic management	
Technique	General anesthesia, rapid sequence induction
Pre-oxygenation	3 minutes
Induction agent	Propofol 120 mg IV
Neuromuscular blocking agent	Rocuronium 50 mg IV
Reversal agent	Sugammadex available (not required)
Neuromuscular monitoring	Unavailable
Laryngoscopy	Cormack-Lehane grade I; no glottic/supraglottic lesions
Endotracheal tube	7 mm cuffed, orotracheal
Maintenance	Isoflurane in oxygen/air mixture (FiO2 0.4)
Post-delivery analgesia	Fentanyl 200 mcg IV
Uterotonic	Oxytocin 15 IU IV infusion
Vasopressors	None required
Antihypertensives	None required
Postoperative course	
Time to emergence	<30 minutes post-surgery
Quality of emergence	Smooth and uneventful
PACU stay	2 hours, stable vital signs
Disposition	Obstetrics ward
Postoperative analgesia	Paracetamol
Neonatal outcome	
Sex/birth weight	Female/3100 g
Apgar scores (1/5/10 min)	9/10/10
Pediatrician at delivery	Yes
Follow-up	
Referrals initiated	Neurology, dermatology, medical genetics

Anesthetic conduct

Given the immediate surgical emergency and the absence of prior neuroimaging or systematic workup, rapid sequence induction was preferred over awake fiberoptic intubation and neuraxial anesthesia. Following pre-oxygenation for three minutes, rapid sequence induction was performed with propofol 120 mg intravenously and rocuronium 50 mg intravenously. Direct laryngoscopy using a standard Macintosh blade yielded a Cormack-Lehane grade I view. No neurofibromatous lesions were identified at the glottic or supraglottic level. Orotracheal intubation was accomplished without difficulty using a 7 mm internal diameter cuffed endotracheal tube. Correct tube placement was confirmed by capnography and bilateral chest auscultation. It should be emphasized, however, that this favorable airway outcome should not be extrapolated to NF1 patients in general. The absence of airway neurofibromas in this case represents a fortunate finding rather than a predictable one, as supraglottic and laryngeal involvement may be clinically silent and undetectable without prior imaging. Consequently, a heightened index of suspicion for difficult airway and preparation for rescue strategies should be maintained in all NF1 patients, regardless of external examination findings.

Anesthesia was maintained with isoflurane in an oxygen/air mixture (fraction of inspired oxygen (FiO2) 0.4). A pediatrician was present at delivery. Intraoperatively, the uterine wall was found to be entirely intact with no evidence of dehiscence or rupture. Following cord clamping and delivery of a female neonate weighing 3100 g with Apgar scores of 9, 10, and 10 at one, five, and 10 minutes, respectively, fentanyl 200 µg was administered intravenously for analgesia. Oxytocin 15 IU was administered by slow intravenous infusion following cord clamping. Hemodynamic parameters remained stable throughout the 45-minute procedure, with no episodes of hypotension or hypertension requiring vasopressor or antihypertensive intervention. Estimated blood loss was below 500 ml.

Emergence from anesthesia was smooth and occurred within 30 minutes of the end of surgery. Extubation was performed without complications. The patient was transferred to the post-anesthesia care unit, where she remained for two hours with stable vital signs before being discharged to the obstetrics ward. Postoperative analgesia was provided with paracetamol. Prior to discharge from the surgical unit, referrals were initiated to medical genetics, neurology, and dermatology for formal NF1 evaluation and systemic workup.

## Discussion

The emergency diagnostic challenge

The clinical diagnosis of NF1 was established at the bedside moments before anesthesia induction, based solely on physical examination findings. This scenario, where a complex multisystem disorder is identified for the first time in the context of an obstetric emergency, places the anesthesiologist in an exceptionally challenging position. The absence of neuroimaging, systemic cardiovascular workup, and pulmonary function data meant that the full extent of the disease burden was unknown. This is not an uncommon situation in resource-limited settings or unbooked obstetric emergencies, and our case contributes to the limited literature addressing the anesthetic management of undiagnosed NF1 in pregnancy [8].

The strikingly extensive tumor burden in our patient raises the question of how such florid disease had remained undiagnosed throughout a 42-year life and a term pregnancy. This underscores a broader concern regarding awareness of NF1 diagnostic criteria among primary care providers and highlights the importance of skin examination across primary care and prenatal settings.

Choice of anesthetic technique

The choice between general and regional anesthesia in NF1 patients is the central clinical dilemma documented across the literature. Regional anesthesia is generally preferred in NF1 when central nervous system involvement can be confidently excluded by preoperative imaging, as it avoids airway manipulation and the hemodynamic consequences of general anesthesia induction [6,7,12]. However, clinically silent spinal neurofibromas have been detected on MRI in up to 40% of asymptomatic NF1 patients in a referral cohort [5], and case reports have documented epidural hematoma following neuraxial techniques in unscreened NF1 patients [4]. In the absence of CT or MRI, the risk of such a complication is unquantifiable and potentially catastrophic.

In our case, the emergency nature of the procedure, the complete absence of prior neuroimaging, and the newly suspected diagnosis of NF1 collectively made general anesthesia the most defensible choice, representing a carefully considered risk-benefit judgment rather than an unequivocally superior option, consistent with the approach reflected in published cases [7,8]. A comparative overview of published obstetric anesthesia cases in NF1 patients is provided in Table 2.

**Table 2:** Published cases of anesthesia in pregnant NF1 patients. Cases were selected to represent the full spectrum of anesthetic management in obstetric NF1. Elective procedures in diagnosed patients with complete preoperative workup demonstrate that both neuraxial and general anesthesia can be safely performed when prior imaging has excluded spinal lesions. Emergency presentations in undiagnosed patients where systematic evaluation was precluded illustrate the decision-making constraints and associated risks when this workup is unavailable, providing the direct comparative context for the present case. CSE: combined spinal epidural; LSCS: lower segment cesarean section; NR: not reported; RSI: rapid sequence induction; CL: Cormack-Lehane

Author (year)	Age	Gestation	NF1 status	Indication	Technique	Key airway/challenges	Outcome
Dounas et al. (1995) [12]	NR	Term	Known	Vaginal delivery	Epidural analgesia	Limited neck extension, Mallampati class II, tracheal deviation	Successful vaginal delivery
Esler et al. (2001) [13]	NR	Term	Undiagnosed	Vaginal delivery	Multiple failed epidural attempts; CSE performed	Not reported	Epidural hematoma-neurological complication
Singh et al. (2014) [6]	26	37 weeks	Known	Elective LSCS (breech + preeclampsia)	Spinal anesthesia	Mallampati III	Successful; T6 block; Apgar 9/10
Çetinkaya Ethemoğlu and Gümüş Özcan (2022) [7]	38	Term	Known	Elective LSCS	General anesthesia	Mallampati II; no oral lesions	Uneventful; smooth emergence
Mallikarjuna et al. (2023) [8]	26	Term	Undiagnosed	Emergency LSCS (meconium)	General anesthesia	Oral neurofibroma present; intraoperative hypertension	Uneventful; Apgar not reported
Present case (2026)	42	40 weeks + 2 days	Undiagnosed	Emergency LSCS (suspected uterine rupture)	General anesthesia, RSI	Mallampati III; plexiform involvement; CL grade I; no glottic lesions	Uneventful; Apgar 9/10/10; smooth emergence <30 min

Airway management

The airway represents the most critical concern in NF1 patients undergoing general anesthesia. Neurofibromas of the tongue, pharynx, larynx, and parapharyngeal space can render laryngoscopy and intubation extremely difficult, and cases requiring emergency tracheostomy have been reported [4]. The risk is particularly elevated when plexiform neurofibromas are present, as these infiltrating tumors grow along the length of nerves and can involve deep anatomical structures including the parapharyngeal space and mediastinum [4]. In our patient, the identification of large lobulated masses involving the breasts and nipple-areolar complexes, consistent with plexiform neurofibromas, raised the clinical concern that similar infiltrating lesions could be present along the airway or in the mediastinum, a possibility that could not be excluded without cross-sectional imaging. In anticipation of a potentially difficult airway, management was guided by current obstetric difficult airway guidelines, emphasizing systematic preparation, a maximum of two laryngoscopy attempts, and clearly defined rescue pathways [14].

Against this background, our patient presented with a Mallampati grade III classification, a thyromental distance of 6 cm, normal mouth opening, and unrestricted neck mobility. The possibility of supraglottic or glottic involvement could not be assessed without indirect laryngoscopy or endoscopy in the emergency context.

Despite these concerns, direct laryngoscopy yielded a Cormack-Lehane grade I view with no lesions identified at the glottic or supraglottic level, and orotracheal intubation was accomplished without difficulty. This favorable outcome reflects both the preparation undertaken and a degree of clinical fortune: the absence of glottic involvement in a patient with such extensive external disease burden cannot be assumed in advance and should never be taken for granted. The full difficult airway cart was immediately available throughout, including video laryngoscopy, supraglottic airway devices, and equipment for surgical airway access.

The choice of rapid sequence induction over an awake fiberoptic approach was deliberate and guided by a careful weighing of competing risks. In a true obstetric emergency with suspected fetal compromise, the time required for awake fiberoptic intubation may itself constitute an unacceptable risk to the fetus and must be balanced against the anticipated degree of airway difficulty. Although the Mallampati grade III classification identified on preoperative assessment would, in isolation, heighten concern for a difficult airway, particularly in the context of NF1, the absence of functional airway compromise was reassuring: there were no stridor, no voice change, and no prior history of difficult intubation. Furthermore, thyromental distance, mouth opening, and neck mobility were all within normal limits. Taken together, these findings supported a decision to proceed with rapid sequence induction, with video laryngoscopy, a supraglottic airway device, and fiberoptic equipment immediately available as rescue options in the event of a failed intubation.

Neuromuscular blockade

The selection of rocuronium over succinylcholine in this case was determined by institutional availability. However, it merits discussion in the context of NF1 pharmacology. Multiple case reports have described increased sensitivity to non-depolarizing neuromuscular blocking agents in NF1, while responses to succinylcholine have been reported as increased, decreased, or normal [4]. Nonetheless, a large retrospective study supports a normal pharmacological response when appropriate monitoring is used, though neuromuscular monitoring is considered mandatory in all NF1 patients receiving relaxants [4]. It should be noted that the evidence base regarding neuromuscular blocker sensitivity in NF1 remains limited and firm conclusions cannot be drawn. Current recommendations uniformly advocate for quantitative neuromuscular monitoring in all NF1 patients receiving neuromuscular blocking agents, given the unpredictability of pharmacological responses in this population.

In our case, monitoring was unavailable, which is a significant limitation we wish to acknowledge explicitly. The decision to proceed with rocuronium was therefore made under suboptimal conditions, and we present it as a pragmatic management decision under emergency resource-limited constraints rather than a recommended approach. Sugammadex was immediately available throughout for reversal if required, providing an important safety margin in the absence of quantitative monitoring. The Obstetric Anaesthetists' Association and Difficult Airway Society (OAA/DAS) obstetric airway guidelines endorse rocuronium with sugammadex availability as an alternative to succinylcholine for rapid sequence induction in obstetric anesthesia [14], and the efficacy and safety of sugammadex for reversal in caesarean section patients have been demonstrated [15]. We recognize, however, that sugammadex availability does not substitute for quantitative neuromuscular monitoring and cannot formally exclude residual blockade. In our case, emergence was smooth and occurred within 30 minutes with no clinical signs of residual blockade, though we acknowledge that clinical assessment alone is insufficient for formal confirmation. We strongly reiterate that neuromuscular monitoring should be considered mandatory whenever feasible in this population.

Hemodynamic stability and cardiovascular considerations

Hypertension in NF1 may result from essential hypertension, renal artery stenosis, or occult pheochromocytoma, the latter being clinically identified in 0.1-5.7% of NF1 patients and representing a potentially lethal perioperative risk if unrecognized [16]. Our patient maintained hemodynamic stability throughout induction, the surgical procedure, and emergence, with no episodes of hypertension or hypotension and no vasopressor support required. While this favorable profile is reassuring, the absence of preoperative urinary catecholamine screening or adrenal imaging means that occult pheochromocytoma cannot be formally excluded and should be addressed as part of the planned postoperative NF1 systemic workup.

Hypothyroidism and anesthetic implications

The patient's history of total thyroidectomy and subsequent hypothyroidism treated with levothyroxine represented an additional perioperative consideration independent of her NF1 diagnosis. Uncontrolled hypothyroidism carries risks of hemodynamic instability, impaired drug metabolism, and prolonged emergence from anesthesia. In our patient, recent laboratory investigations confirmed euthyroid status on her current regimen of levothyroxine 100 micrograms daily, and her perioperative course was entirely consistent with adequate thyroid replacement: emergence was prompt, occurring within 30 minutes, and hemodynamic parameters were stable throughout.

Obstetric and neonatal outcome

The obstetric indication in this case, clinical suspicion of uterine rupture based on uterine hypertonicity and hyperalgesia, was not confirmed intraoperatively, with the uterine wall found to be completely intact. Nevertheless, the clinical decision to proceed with emergency cesarean section was appropriate given the potential consequences of a missed uterine rupture in a term primigravida. The neonatal outcome was excellent, with Apgar scores of 9, 10, and 10 at one, five, and 10 minutes and a birth weight of 3100 g, with a pediatrician present at delivery.

From an NF1-specific obstetric perspective, pregnancy is associated with an increase in the number and size of neurofibromas [4], as well as elevated rates of hypertension, HELLP syndrome, and preterm labor [3,4]. Advanced maternal age at first pregnancy, as in our patient, adds a further layer of obstetric risk. In this case, neither NF1 nor advanced maternal age adversely affected the perioperative course or neonatal outcome.

Limitations

This case report has several limitations that should be acknowledged. First, the diagnosis of NF1 was established clinically at the bedside without formal genetic confirmation, and histopathological verification of the suspected plexiform neurofibromas was not obtained in the perioperative period. Second, the absence of preoperative neuroimaging means that spinal or intracranial neurofibroma involvement cannot be excluded retrospectively, and the true extent of central nervous system disease burden remains unknown. Third, airway endoscopy was not performed, and the absence of glottic or supraglottic lesions was established only at direct laryngoscopy under general anesthesia rather than by prior assessment. Fourth, quantitative neuromuscular monitoring was unavailable, and the adequacy of neuromuscular recovery was assessed clinically rather than instrumentally. Fifth, postoperative follow-up data are limited, and the results of the planned referrals to medical genetics, neurology, and dermatology are not available at the time of this report.

## Conclusions

This case illustrates that safe anesthetic management of undiagnosed NF1 in an obstetric emergency is achievable through systematic clinical recognition, anticipatory airway preparation, and informed technique selection in the absence of neuroimaging. General anesthesia with full difficult airway preparedness represents the most defensible approach when central nervous system involvement cannot be excluded. Quantitative neuromuscular monitoring should be considered mandatory whenever feasible. Postoperative multidisciplinary referral is essential, as the full extent of multisystem involvement requires characterization outside the emergency context. The recurrence of this presentation across different healthcare settings underscores the need for greater NF1 awareness in primary and antenatal care.
